# Epidemiological Characteristics and Trend in the Incidence of Human Brucellosis in Iran from 2009 to 2017

**DOI:** 10.34172/jrhs.2021.70

**Published:** 2021-12-05

**Authors:** Faezeh Norouzinezhad, Hossein Erfani, Abbas Norouzinejad, Fatemeh Ghaffari, Farzad Kaveh

**Affiliations:** ^1^Department of Nursing and Midwifery, Ramsar Fatemeh Zahra School of Nursing and Midwifery, Babol University of Medical Sciences, Mazandaran, Iran; ^2^Ministry of Health and Medical Education, Tehran, Iran

**Keywords:** Epidemiology, Human brucellosis, Infectious diseases, Iran, Malta fever

## Abstract

**Background:** The development of preventive measures and promotion of healthcare delivery systems for disease management is dependent on detecting the incidence rates of the diseases and important risk factors. The current study aimed to determine some epidemiological indices and trends of the human brucellosis incidence in Iran between 2009 and 2017.

**Study design:** A descriptive cross-sectional study.

**Methods:** In the current study, online data were gathered from all cases of brucellosis who were potentially or definitely diagnosed and registered in medical centers, hospitals, laboratories, and private clinics in all districts of Iran. Epidemiologic questionnaires were used to collect data on demographic and background characteristics, risk factors, high-risk behaviors, and lab test results, respectively, based on the national brucellosis control plan.

**Results:** A total of 138,448 cases of brucellosis have been studied in Iran from 2009 to 2017. The highest (25.89) and the lowest (12.07) incidence rates were observed in 2014 and 2009, respectively. In this nine-year study, the highest incidences were reported in the Hamadan, Lorestan, Kurdistan, and Kermanshah provinces. The cumulative percentages of the disease were estimated for different variables at the following rates: by gender: 57.9% for males; by age groups: 36.2% and 16.7% for the 25-44 and16-24 years age groups, respectively; by occupation: 33.2% for housewives; and by residential area: 75.7% for rural residents.

**Conclusions:** Based on the obtained results, although the incidence of human brucellosis in Iran has declined since 2015, the number of cases has remained high.

## Introduction

*B rucella* is a small gram-negative, facultative intracellular, severely aerobic, and resilient bacteria that cause brucellosis (zoonosis) in cows, sheep, goats, and humans^1^. Based on differences in the main hosts and their pathogenicity, *Brucel la* is classified into six species. *B. abortus* causes bovine brucellosis that gives rise to undulant fever (brucellosis) in humans. However, this disease can also be caused by *B. melitensis*, *B. suis,* and *B. canis*^2,3^. Human brucellosis presents a wide range of clinical symptoms and is difficult to diagnose due to its similarity to other diseases^4^. Brucellosis poses an occupational hazard to those in contact with infected animals. A non-occupational source of this disease can be the consumption of fresh unpasteurized dairy products ^5^. Other than its serious physical complications, this disease is one of the most challenging factors for economic development in many countries. Upon acquiring the disease, the patients become dependent on others for performing their daily activities due to body weakness which imposes a financial burden on the patient, family, and healthcare system^6^.

The annual reported incidence of new cases of brucellosis is 500,000 cases globally. The prevalence of brucellosis in some countries exceeds 10 cases per 100,000 population. Nevertheless, the reported incidence is lower than the actual rate since for every reported case, there are 26 undiagnosed cases. In endemic regions, the incidence rate ranges from less than 0.03 to more than 200 per 100,000 population. Many regions in the world, such as the Middle East (including Iran), Africa, Latin America, Central Asia, and the Mediterranean region are still considered the endemic areas for brucellosis^7^. In terms of the incidence of brucellosis, Iran ranks fourth in the world and first in the Eastern Mediterranean region ^7,8^. One systematic review reported an annual incidence of brucellosis among Middle Eastern countries ranging from 0.73 to 149.54 per 100,000 population. The results of a study conducted in Iran by Mirzanejad et al. (2017) indicated that the incidence of brucellosis ranged from 7.00 to 276.42 per 100,000 population over 18 years^9^.

Brucellosis has become a health priority in different regions of Iran due to the outcomes of the disease on public health and the patient’s social functioning and numerous problems in controlling the disease ^10,5^. The development of preventive measures and promotion of healthcare delivery systems for disease management depends on detecting the incidence and prevalence rates as well as important risk factors of brucellosis.

 The current study was conducted to determine some of the epidemiological indices and the trend of human brucellosis incidence in Iran between 2009 and 2017.

## Methods

 The current study was a descriptive cross-sectional one. All cases of brucellosis that were potentially or definitely diagnosed were gathered online from medical centers, hospitals, laboratories, and private clinics in all districts of Iran.

 Data were collected via epidemiologic questionnaires, based on the National Brucellosis Control Plan. These questionnaires consisted of three parts:

 The first part included items related to demographic and background characteristics, such as age, gender, occupation, and residential area.

 The second part included items on risk factors and high-risk behaviors, including the history of contact with livestock, history of livestock vaccination, history of unpasteurized dairy products consumption, type of contact with livestock in the past 18 months, and month of being infected with the disease.

 The third part involved items on lab test results, such as the results of the Wright, Coombs Wright, and 2-Mercaptoethanol (2-ME) tests.

 The procedure was as follows:

1. Suspected cases (based on epidemiologic evidence and disease symptoms) were identified and referred to the laboratory by a physician.
2. Serum samples from the referred cases were prepared in each provincial lab. The Rose Bengal Plate Test (RBPT), as the most common screening test for brucellosis, was first performed on the samples. Next, Wright’s Standard Tube Agglutination Test was performed on samples that were positive by RBPT to confirm the result and determine the titer. Eventually, the 2-ME test was performed on every sample that tested positive in both the RBPT and Wright’s test to measure the immunoglobulin G (IgG) titer as an indicator of the active phase of brucellosis. Following the National Brucellosis Control Plan, a titer of ≥1.40 in the 2-ME test was used as the diagnostic criteria for brucellosis in this study^11^. The laboratory data included in the analysis were based on the Wright and 2-ME tests performed on the serum samples and extracted as titers.
3. The patients’ characteristics were registered by physicians in the questionnaires with items on demographic and background characteristics, risk factors, and high-risk behaviors, and then reported to the district health center.
4. All data were registered in the brucellosis report form that was available in all urban health centers and sent to the district health centers through an online portal system.
5. Complementary data were registered in the epidemiologic examination sheets in the portal system of the district health centers. The data were then sent to the Deputies of Health in the provincial universities of medical sciences.
6. The disease cases were reported to the Communicable Diseases Center (CDC) of the Ministry of Health and Medical Education by the aforementioned Deputies of Health in each provincial medical university.
7. The data collected and registered through the previous steps were acquired by the researchers after obtaining the permission from the CDC authority. The data were analyzed using SPSS software (Version 18) and reported afterward. The data were analyzed using descriptive statistical methods and presented by frequencies and percentages. The incidence rates were estimated and reported (yearly) per every 100,000 persons in populations at risk.

The permission for using the national database and records was granted by the Iranian Center for Communicable Diseases Control, Ministry of Health and Medical Education, Tehran, Iran (NO: 304/1309).

## Results

 A total of 138,448 cases of brucellosis have been studied in Iran from 2009 to 2017.

The cumulative estimated percentages of disease for different variables were as follows: by gender: 57.9% for males; by age: 36.2% and 16.7% for the 25-44 and 16-24-year age groups; and by residential area: 75.7% for rural residents. The cumulative percentages of disease cases based on a positive history of livestock vaccination were 42.2%, 48.8% among those who kept their livestock near their residential area in the previous 18 months, and 28.5% for the consumption of unpasteurized dairy products, such as milk and cheese. Furthermore, throughout the study years, the participants reported a history of contact with livestock (Table 1).

**Table 1:** Distribution of the relative frequencies of the population with brucellosis based on the studied variables

Variable	**2009**		**2010**		**2011**		**2012**		**2013**		**2014**		**2015**		**2016**		**2017**	
	**Number**	**Percent**	**Number**	**Percent**	**Number**	**Percent**	**Number**	**Percent**	**Number**	**Percent**	**Number**	**Percent**	**Number**	**Percent**	**Number**	**Percent**	**Number**	**Percent**
Gender																		
Male	4876	55.1	5811	55.4	7246	56.9	9238	57.6	11446	59.9	11869	58.8	11556	58.4	8970	58.4	9157	57.7
Female	3969	44.9	4674	44.6	5501	43.1	6360	39.7	7349	38.5	8327	41.2	8247	41.6	6384	41.6	6705	42.3
Age																		
<15	1153	13	1528	14.6	1758	13.8	2173	13.6	2585	13.5	2738	13.6	2397	12.1	1811	11.8	1915	12.1
16 - 24	182	20.7	2098	20	2545	19.9	2960	18.5	3449	18.1	3383	16.8	2910	14.7	2036	13.3	1916	12.1
25 – 44	2965	33.5	3467	33.1	4332	33.9	5447	34	6511	34.1	7701	38.1	7696	38.9	5889	38.4	6063	38.2
45 - 54	1370	15.5	1590	15.2	1938	15.2	2126	13.3	2585	13.5	2935	14.5	3152	15.9	2579	16.8	2639	16.6
55<	1530	17.3	1802	17.2	2192	17.2	2509	15.6	2958	15.5	3439	17	3648	18.4	3039	19.8	3329	21
Residential area																
Rural	6898	78	8099	77.2	10250	80.3	12472	77.8	14482	75.8	15799	78.2	15135	76.4	11404	74.3	1879	74.9
Urban	1947	22	2386	22.8	2515	19.7	3252	20.3	4070	21.3	4214	20.9	4418	22.3	3464	122.6	3667	23.1
Nationality																	
Iranian	8785	99.3	10395	99.1	12631	66.1	15877	83.1	18934	99.1	20022	99.1	19643	97.3	15212	99.1	15660	98.7
Afghan	58	0.7	86	0.8	130	0.7	157	0.8	165	0.9	165	0.8	145	0.7	129	0.8	181	1.1
Pakistani	1	0	2	0	1	0	-		2	0	-		-		-		12	0.1
Iraqi	1	0	2	0	2	0	1	0	-		6	0	10	0	7	0	-	
History of contact with livestock													
No	2248	25.4	1896	18.1	2047	16	2334	14.6	2889	15.1	4052	20.1	2436	12.3	2177	14.2	2585	16.3
Yes	6179	69.9	7929	75.6	10427	81.7	12392	77.3	14664	76.8	16144	79.9	15559	78.6	11721	76.3	11915	75.1
History of livestock vaccination													
Yes	2301	26	5112	48.8	7153	56	8459	52.8	10405	54.5	7393	36.6	7340	37.1	5136	33.51	5380	33.9
No	814	9.2	2533	24.2	2968	23.3	3881	24.2	4672	24.5	12803	63.4	5168	26.1	3648	23.8	3799	24
Unclear	18	0.2	-		-		616	3.8	997	5.2	-		2852	14.4	2789	18.2	3079	19.4
History of consuming unpasteurized dairy products										
Milk	3516	39.8	3244	30.9	4317	33.8	219	1.4	30501	18.3	4276	21.21	5914	29.9	4459	29	4756	30
Cheese	1063	12	1205	11.5	1403	11	39	0.2	1296	6.8	1310	6.5	1587	8	1162	7.6	1168	7.4
Whipped cream	69	0.8	68	0.6	113	0.9	5	0	95	0.5	44	0.2	88	0.4	59	0.4	69	0.4
Butter	38	0.4	59	0.6	92	0.7	4	0	78	0.4	97	0.5	104	0.5	75	0.5	59	0.4
Colostrum	91	1	160	1.5	190	1.5	7	0	225	1.2	551	2.7	299	1.5	196	1.3	248	1.6
Kaymak	95	1.1	264	2.5	138	1.1	48	0.3	170	0.9	153	0.8	123	0.6	166	1.1	225	1.4
Milk and cheese	3122	35.3	3748	35.7	4410	34.5	284	1.8	4113	21.5	7407	36.7	6346	32	4918	32	5058	31.9
Ice cream	45	0.5	87	0.8	115	0.9	5	0	117	0.6	155	0.8	202	1	139	0.9	246	1.6
None	174	2	-		-		-		-		-		-		-		-	
Unclear	231	2.6	-		-		-		-		-		39	0.2	-		33	0.2
Type of contact with livestock during the last 18 months									
Keeping the animal near one’s residential area	304	3.4	1774	16.9	5617	44	9362	58.4	12157	63.6	10382	51.4	13483	68.1	9666	63	4859	30.6
Contact with live animals	3756	42.5	3206	30.6	1447	11.3	1194	7.4	2235	11.7	3217	15.9	13837		1658	10.8	5724	36.1
Contact with birth secretions and/or the aborted fetus	682	7.7	1210	11.5	2121	16.6	12.6	7.5	-		-		-		-		34	0.2
Slaughtering the animal	100	1.1	659	6.3	887	6.9	380	2.4	-		-		326	1.6	257	1.7	1128	7.1
Contact with the animal corpse and its secretions after its slaughtering	507	5.7	708	6.8	42	0.3	56	0.3	305	1.6	1999	7.9	218	1.1	36	0.2	69	0.4

**Figure 1:**
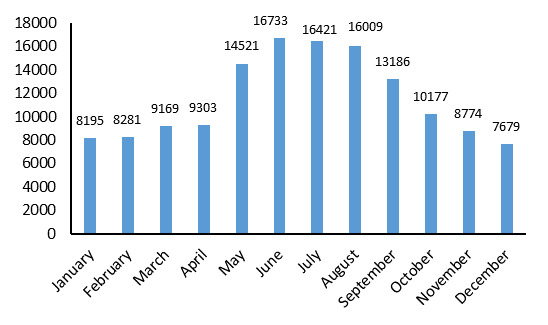
The trend in the numbers of cases of brucellosis according to the month of disease presentation

In most of the years studied, the highest numbers of cases of brucellosis were observed in June, July, and August (Figure 1).

 Based on the results of the present study, the highest number of brucellosis cases during the nine-year study period occurred among farmer-ranchers (4,196), housewives (6,503), and students (2,253), in 2011, 2015, and 2014 respectively.

 Based on Wright’s serology, examination of the brucellosis frequency showed that most patients had titers of 1.160 and 1.320 by the Wright and Coombs’ Wright tests (positive cut-off≥1:160), respectively. Furthermore, most patients had titers of 1.80 for the 2-ME test.

**Figure 2:**
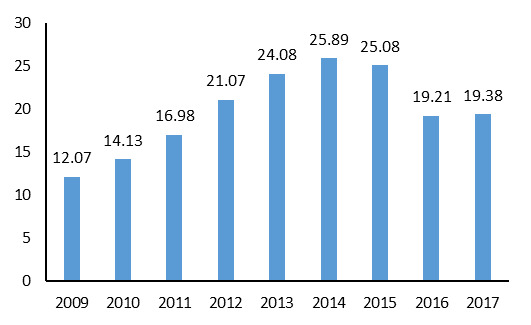
The trend of brucellosis incidence in 100,000 population, 2009-2017

**Figure 3:**
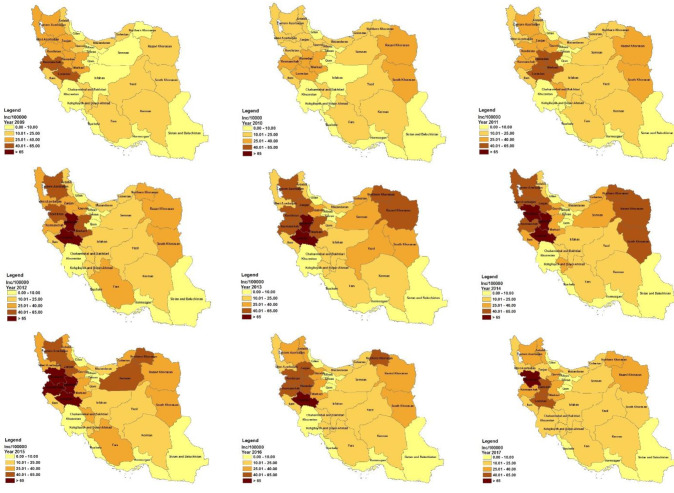
Geographical distribution of brucellosis in Iran between 2009 and 2017.

The highest frequency (72.8%) of individuals seropositive for brucellosis by the 2-Mercaptoethanol test was observed in 2016. The highest (25.89 per 100,000 population) and lowest (12.07 per 100,000 population) incidence rates were observed in 2014 and 2009, respectively. Based on the nine-year examination of brucellosis, the highest incidence rates were observed in the Hamedan, Lorestan, Kordestan, and Kermanshah provinces (Figure 2, Table 2). The geographical distribution of brucellosis in Iran between 2009 and 2017 is illustrated in Figure 3.

**Table 2:** Frequency and incidence rate of human brucellosis by province

	**2009**		**2010**		**2011**		**2012**		**2013**		**2014**		**2015**		**2016**		**2017**	
Province	**Number**	**Incidence rate**	**Number**	**Incidence rate**	**Number**	**Incidence rate**	**Number**	**Incidence rate**	**Number**	**Incidence rate**	**Number**	**Incidence rate**	**Number**	**Incidence rate**	**Number**	**Incidence rate**	**Number**	**Incidence rate**
Eastern Azerbaijan	937	31.27	750	24.68	1063	34.50	1767	56.69	1785	56.61	1628	51.03	1426	44.18	1082	33.13	1237	37.31
Ardabil	210	16.93	185	14.87	186	14.89	183	14.60	255	20.28	253	20.05	506	39.98	428	33.68	440	33.18
Isfahan	62	1.30	366	7.60	511	10.47	653	13.25	954	19.17	835	16.62	807	15.91	763	14.89	722	13.57
Khuzestan	-	-	387	8.64	333	7.34	337	7.37	439	9.61	316	6.81	343	7.34	282	5.98	542	10.87
Ilam	116	21.16	93	16.75	115	20.62	126	22.41	135	23.83	151	26.44	139	24.15	109	18.78	129	20.87
Mazandaran	316	10.86	441	14.77	435	14.15	565	18.14	522	16.54	356	11.13	412	12.71	422	12.85	450	13.69
Bushehr	3	.3	10	.9	28	2.71	31	2.93	33	3.04	56	5.04	36	3.16	42	3.61	34	3.11
South Khorasan	141	21.63	244	37.13	194	29.28	172	25.19	248	35.26	415	57.28	235	31.49	197	25.62	144	19.79
West Azerbaijan	108	27.45	626	16.92	797	21.39	1315	34.96	563	14.82	1696	44.23	1325	34.22	934	23.88	739	21.79
Fars	580	12.91	664	14.61	944	20.53	1302	28.02	1166	24.82	1165	24.54	1248	26	970	19.99	850	17.15
Tehran	186	1.57	160	1.33	185	1.51	248	2	317	2.51	348	2.71	531	4.07	381	2.87	327	2.01
Kerman	520	18.43	451	15.66	421	14.32	476	15.95	580	19.15	509	16.56	599	19.21	502	15.86	579	18.44
Chaharmahal and Bakhtiari	104	11.81	135	15.21	257	28.70	223	24.62	201	21.94	221	23.86	275	29.35	135	14.24	137	13.84
Northern Khorasan	60	7.10	136	15.80	206	23.74	256	29.53	347	40.06	404	46.69	402	46.51	380	44.2	326	35.47
Sistan and Baluchistan	72	2.90	188	7.49	154	6.07	112	4.33	162	6.16	98	3.66	82	3	92	3.31	161	5.22
Razavi Khorasan	1208	20.72	1702	28.79	1957	32.64	2143	35.24	2606	42.26	2508	40.10	2231	35.17	1900	29.52	2608	39.30
Semnan	49	7.97	97	15.58	117	18.53	125	19.38	220	33.40	209	31.06	284	41.32	138	19.46	133	19.33
Kermanshah	841	43.83	721	37.32	773	39.73	746	38.29	1058	54.31	1241	63.66	1381	70.79	841	43.07	734	38.33
Qazvin	200	16.98	198	16.64	313	26.04	322	26.48	362	29.43	448	36	494	39.24	411	32.26	349	26.9
Qom	40	3.60	74	6.55	79	6.85	74	6.27	142	11.77	92	7.45	158	12.51	80	6.19	37	2.86
Golestan	263	15.46	285	16.39	199	11.19	344	19.16	590	32.54	525	28.66	507	27.40	447	23.92	364	18.48
Gilan	40	1.63	69	2.79	66	2.66	76	3.05	66	2.63	60	2.39	92	3.65	95	3.75	78	2.95
Lorestan	831	47.79	638	36.53	845	48.16	1159	66.02	1277	72.69	1759	100.06	2035	115.68	1420	80.65	1176	62.33
Markazi	461	33.20	444	31.68	599	42.36	735	51.87	596	41.97	510	35.84	542	38.09	432	30.22	437	28.40
Alborz	-	-	26	11.13	77	3.19	84	3.40	162	6.40	136	5.25	138	5.20	131	4.82	242	8.74
Kordistan	415	28.21	354	23.86	536	36.64	762	51.15	976	64.33	1584	102.52	1319	83.83	972	60.63	1250	80.16
Kohgiluyeh and Boyer-Ahmad	116	17.88	59	9.02	61	9.26	68	10.16	88	12.94	191	27.65	130	18.52	146	20.47	111	15.8
Hormozgan	32	2.12	37	2.40	17	1.07	22	1.36	28	1.69	18	1.06	15	.86	29	1.63	25	1.42
Hamedan	436	25.12	586	33.55	835	47.48	1144	65.20	1498	85.57	1681	96.23	1305	74.87	882	50.74	894	50.44
Yazd	142	13.65	130	12.29	170	15.82	135	12.42	281	25.55	166	14.92	215	19.10	154	13.52	171	13.81
Zanjan	256	25.73	229	22.78	290	28.55	312	30.47	446	43.21	617	59.30	591	56.35	549	51.91	435	40.02
Total	8845	12.07	10485	14.13	12765	16.98	16035	21.07	19103	24.80	20196	25.89	19803	25.08	15354	19.21	15862	19.38

## Discussion

 A total of 138,448 cases of brucellosis were observed over the nine-year study period. The incidence rate of the disease ranged from 12.07 to 25.89. An increasing trend was observed until 2014, which may have been due to the public’s lack of awareness of preventive measures, consequences of consuming unpasteurized dairy products, lack of livestock vaccination, issues related to the statistical system, and absence of accurate registration of data in the portal system for diseases during these years.

Esmaeili et al. suggested that a number of cases were undiagnosed, particularly in poor and remote provinces. However, the performance of the health surveillance system has improved in recent years in Iran. With the expansion of the healthcare system, one would expect the number of reported cases to increase ^12^. Nevertheless, the results of the present study indicates a declining trend in the disease from 2015 until 2017. This decline may be attributed to increased awareness about the modes of disease transmission and preventive measures through the education of ranchers and those in contact with raw livestock products. Furthermore, the increased vaccination coverage of lambs and care of infected and suspected cases of livestock can contribute to the prevention of disease spread in the community. Compared to the incidence rates of brucellosis in some countries (not exceeding 10 cases per 100,000 population), the results of this study indicate that the disease is endemic in Iran. In endemic regions, the incidence rate ranges from 0.03 to 200 cases per 100,000 population. However, for every reported case there may be 26 undiagnosed cases indicating that the incidence rate of the disease may be much higher^13,14^. Based on the results of the study conducted by Rostami et al. (2015) on 1,698 patients across 30 provinces in Iran, the estimated mean incidence of brucellosis was 29.83 per 100,000 population ^15^. Despite the observation of a declining trend in recent years, the geographical location of Iran and its adjacency to endemic countries, such as Iraq, Pakistan, and Afghanistan are important risk factors for the re-emergence and spread of brucellosis. Although high-quality veterinary services have been delivered to these countries to control animal diseases, the danger of the disease spreading from country to country still persists ^12^.

Based on the results of the present study, the rate of brucellosis has remained high in Iran in recent years (at least in some provinces, such as Razavi Khorasan and Kordestan). Inaccurate laboratory diagnostics might be an important reason for the persistently high rate of brucellosis in Iran. False-negative cases during culturing, tubal agglutination, and the higher antibody titers among ordinary people in endemic regions have led to the reduced efficiency of these methods. The failure to use molecular methods or other diagnostic techniques in many medical centers and laboratories across the country further complicates the diagnosis of the disease. Other than the absence of appropriate and alternative verification methods, there are limiting diagnostic factors, such as the low quality of kits available in the market and the absence of a comprehensive seroepidemiologic study that can determine the diagnostic level of specific antibodies through serological techniques ^16,17^.

The results of this nine-year study indicated the highest incidence rates in the provinces of Hamedan, Lorestan, Kordestan, and Kermanshah in Iran. The classification conducted in 2010 showed that among all provinces, Kermanshah, Lorestan, Kordestan, and Hamedan had the highest incidence rates of brucellosis. These provinces fall in the ‘very high’ category ^13,18^, indicating that, despite the steps taken to control the disease, the incidence rates have remained high. Health policy-makers need to plan for public education (particularly during months in which the probability of incidence is higher), expand livestock vaccination, continually assess disease control programs and follow-ups, and address administrative issues.

According to the obtained results, the disease is mostly present in males. This finding is concordant with those of other studies conducted across Iran’s provinces ^15,11-21^. Nevertheless, throughout the years of this study, the percentages of cases of disease remained similar in both genders. In this respect, Azizi et al. (2010) believe that since women work alongside men in such occupations as ranching and farming, the number of cases is high among women as well. Moreover, women are in contact with the causative agent and can inhale it while milking the animals and cleaning their shelters^22^. Incorrect habits of food consumption among the Iranian society, such as the consumption of meat products and offal with insufficient cooking due to inadequate information on the routes of disease transmission may also contribute to the increase in the number of brucellosis cases in both genders.

The highest percentages of cases were observed in the 16-24 and 25-44 years age groups. This may be explained by these age groups’ occupational exposures and involvement in ranching, farming, butchering, and slaughtering livestock. Most studies conducted in Iran have also reported the highest number of brucellosis cases among adolescent and young adult age groups ^18,2,2^. According to Zeinalian Dastjerdi et al. (2012), given the risk of contact with infected animals and their products, the mean age of the affected cases was 31.3 years^23^. Nevertheless, other studies have reported a peak of cases in the age group older than 50 years ^20^.

In this study, the most common mode of infection was through the consumption of unpasteurized dairy products, such as milk and cheese. Keeping livestock in residential areas and contact with live animals were the main routes of transmission. Based on numerous reports, the dominant route of *Brucella* transmission to humans is through the digestive tract (i.e., the consumption of unpasteurized dairy products, such as fresh milk and other milk-derived products, particularly whipped cream and ice cream, which contain high levels of the bacteria)^13^. Moreover, consistent with the literature, the highest number of cases were observed in rural areas^12,24-25^.

The highest numbers of cases were observed in the second and third months of spring and the first month of summer. According to Golshani et al. (2017), the incidence of brucellosis increases during spring and summer as the ranchers are in direct contact with aborted fetuses, and infected dairy products are consumed ^10^. The global seasonal pattern of brucellosis indicates that the disease is more common in the first half of the year, during the animal reproduction period ^24^. The seasonal pattern of brucellosis in Iran was also assessed in a meta-analysis, which reported the highest prevalence in the spring and summer and the lowest prevalence during the autumn and winter months ^24^.

It was observed that housewives were most affected by the disease since they worked alongside men and were responsible for milking animals. Moreover, they lived in a contaminated environment and inhaled the causative agent due to the adjacency of their residential areas to animal shelters, which exposed them to the disease. Farmer-ranchers have the second most common occupation affected by brucellosis. Brucellosis is usually considered an occupational disease, as it is most commonly observed among slaughterhouse workers, veterinary doctors, laboratory technicians, hunters, farmers, and ranchers ^26^.

## Conclusions

Although brucellosis has witnessed a declining trend in Iran since 2015, the number of infected cases has remained high. Given the high number of recurrent cases^27^, changes in the epidemiologic pattern of the disease, and clinical factors, research should focus on the epidemiologic cases of the disease in different regions of Iran. Male gender, adolescent and young adult age groups, housewives and farmer-rancher occupations, and warm seasons of the year are probable risk factors for brucellosis in humans. Advanced surveillance systems that are focused on probable risk factors can effectively protect against the disease. Comprehensive measures need to be taken to control the routes of disease transmission from domesticated animals and their products to humans. Preventive measures must also be taken by health officials and health service providers. In this regard, the following measures are recommended:

 Frequent visits must be made to restaurants and dining halls across the city as well as various centers responsible for the distribution, storage, and supply of raw livestock products, such as centers providing red and white meat, chain stores, centers supplying poultry products, and butcheries.

The *Brucella* test must be conducted on animals and positive cases must be slaughtered in industrial and semi-industrial cattle farms.

 Given the high number of cases among men and housewives, educating these people on disease prevention in contact with animals can help control the disease. Such measures as the provision of education on the significance of the correct handwashing procedure with soap and water, application of gloves and face masks in contact with affected livestock and when cleaning the affected livestock’s shelter, and proper air conditioning can all prevent infection in these individuals.

 Given the increased number of cases during the spring and summer months, education, therapeutic interventions, and screening should be increased during the seasons in which disease transmission is higher.

 The culture of consuming pasteurized dairy products should be institutionalized in society to achieve this goal. The Department of Education and Health Networks can play significant roles in schools and across urban and rural areas, respectively.

 The provision of continuous education through health service providers and mass media campaigns are required to raise awareness about the modes of disease transmission, symptoms, and methods of disease prevention.

 The expansion of intersectoral collaborations among veterinary clinics, governorates, prefectures, village administrators, non-governmental organizations, teachers, and all classes of society (particularly journalists, etc.) are strategies to reduce the incidence of the disease. Education on the significance of vaccinating livestock and the introduction of centers providing this service, especially to rural inhabitants, are indicated as well.

## Acknowledgments

 The authors would like to thank the personnel at the urban and rural health centers and all those who helped to register and collect the data.

## Conflict of Interest

 The authors declare that they have no conflict of interest regarding the publication of this study.

## Funding

 This study was not financially supported by any governmental, private, or non for profit organization or institute.

## Authors’ contributions

 F.GH. and F.N. wrote the manuscript; H.E., A.N., and F.K. collected the data and reviewed/edited the manuscript; F.GH. and F.N. contributed to the discussion section and reviewed/edited the manuscript, and F.GH. collected the data and contributed to the discussion section.

 Highlights

- A total of 138,448 cases of brucellosis were studied in Iran from 2009 to 2017.
- The highest (25.89) and the lowest (12.07) incidence rate was observed in 2014 and 2009, respectively.
- The highest incidence rates were reported in Hamadan, Lorestan, Kurdistan, and Kermanshah provinces.
